# Revealing the characteristics of SETD2-mutated clear cell renal cell carcinoma through tumor heterogeneity analysis

**DOI:** 10.3389/fgene.2024.1447139

**Published:** 2024-07-25

**Authors:** Shansen Peng, Zhouzhou Xie, Huiming Jiang, Guihao Zhang, Nanhui Chen

**Affiliations:** ^1^ Meizhou Clinical Institute of Shantou University Medical College, Meizhou, China; ^2^ Department of Urology, Meizhou People’s Hospital, Meizhou Academy of Medical Sciences, Meizhou, China

**Keywords:** clear cell renal cell carcinoma, ScRNA-seq, SETD2, macrophage, prognosis

## Abstract

**Background:**

Renal cell carcinoma (RCC) is the most prevalent type of malignant kidney tumor in adults, with clear cell renal cell carcinoma (ccRCC) comprising about 75% of all cases. The SETD2 gene, which is involved in the modification of histone proteins, is often found to have alterations in ccRCC. Yet, our understanding of how these SETD2 mutations affect ccRCC characteristics and behavior within the tumor microenvironment is still not fully understood.

**Methods:**

We conducted a detailed analysis of single-cell RNA sequencing (scRNA-seq) data from ccRCC. First, the data was preprocessed using the Python package, “scanpy.” High variability genes were pinpointed through Pearson’s correlation coefficient. Dimensionality reduction and clustering identification were performed using Principal Component Analysis (PCA) and the Leiden algorithm. Malignant cell identification was conducted with the “InferCNV” R package, while cell trajectories and intercellular communication were depicted using the Python packages “VIA” and “cellphoneDB.” We then employed the R package “Deseq2” to determine differentially expressed genes (DEGs) between groups. Using high-dimensional weighted gene correlation network analysis (hdWGCNA), co-expression modules were identified. We intersected these modules with DEGs to establish prognostic models through univariate Cox and the least absolute shrinkage and selection operator (LASSO) method.

**Results:**

We identified 69 and 53 distinctive cell clusters, respectively. These were classified further into 12 unique cell types. This analysis highlighted the presence of an abnormal tumor sub-cluster (MT + group), identified by high mitochondrial-encoded protein gene expression and an indication of unfavorable prognosis. Investigation of cellular interactions spotlighted significant interactions between the MT + group and endothelial cells, macrophaes. In addition, we developed a prognostic model based on six characteristic genes. Notably, risk scores derived from these genes correlated significantly with various clinical features. Finally, a nomogram model was established to facilitate more accurate outcome prediction, incorporating four independent risk factors.

**Conclusion:**

Our findings provide insight into the crucial transcriptomic characteristics of ccRCC associated with SETD2 mutation. We discovered that this mutation-induced subcluster could stimulate M2 polarization in macrophages, suggesting a heightened propensity for metastasis. Moreover, our prognostic model demonstrated effectiveness in forecasting overall survival for ccRCC patients, thus presenting a valuable clinical tool.

## 1 Introduction

Renal cell carcinoma (RCC), which primarily originates from renal tubule epithelial cells, is the most common form of malignant kidney tumor among adults (Hsieh et al., 2017). Specifically, clear cell renal cell carcinoma (ccRCC), represents about 75% of all RCC cases (Siegel et al., 2020). While surgical interventions offer limited improvement to overall patient survival, the prognosis remains dire due to the disease’s few early symptoms, the varied efficacy of targeted treatment options, and the current lack of relevant biomarkers (van der Mijn et al., 2014; Sánchez-Gastaldo et al., 2017; Siegel et al., 2020). Considering these factors, it is crucial to investigate the key factors and possible mechanisms leading to metastasis in ccRCC progression.

The defining molecular characteristic of ccRCC involves the dysfunction of the von Hippel-Lindau (VHL) tumor suppressor *via* several processes (Barata and Rini, 2017). Essentially, VHL acts as a regulator of the cell’s response to oxygen availability by interacting with hypoxia-inducible factor-1 (HIF-1) (Wiesener et al., 2001). However, VHL’s dysfunction in ccRCC results in an excess of HIF-1, which in turn activates genes connected to tumor metabolism and angiogenesis (Barata and Rini, 2017). Yet, experiments have shown that VHL deficiencies alone do not cause tumors in mice, suggesting that other factors play a role in ccRCC’s initiation and progression (Haase et al., 2001).

We note frequent mutations in several other genes in close genomic proximity to VHL, including PBRM1, SETD2, BAP1, and KDM5C (Cotta et al., 2023). The SETD2 gene, inactivated in 8%–30% of ccRCC cases, alters the transcription, DNA damage repair, and selective splicing through its regulation of H3K36 trimethylation (H3K36me3) (Mano et al., 2021; Walton et al., 2023). Its mutation in ccRCC results in increased genome chromatin accessibility and the potential for cancerous transcription (Ho et al., 2016; Xie et al., 2022). However, despite this understanding, SETD2-mutated ccRCC’s behavior in the actual tumor environment remains unclear.

Our study will employ single-cell RNA sequencing (scRNA-seq) data to examine primary ccRCC samples retrieved from clinical contexts. Our objective is to identify a particular subtype of ccRCC found to highly express mitochondrial-encoded protein genes. Subsequently, we aim to verify their origin from SETD2-mutated ccRCC. In addition, we will delve into their transcriptomic expression patterns and understand their biological characteristics within the tumor microenvironment. The ultimate goal of our research is to develop a prognostic model based on the identified subtype, which can then be used in clinical prognoses.

## 2 Materials and methods

### 2.1 Pre-treatment of samples

scRNA-seq data labeled GSE178481 was gathered from the Gene Expression Omnibus (GEO) database (https://www.ncbi.nlm.nih.gov/geo/). This includes primary ccRCC and corresponding peritumoral tissue data from nine patients. The TCGA-KIRC cohort was sourced from The Cancer Genome Atlas (TCGA) database (https://portal.gdc.cancer.gov/). After removal of samples with incomplete datasets, 532 KIRC patients remained in our study scope. The E-MTAB-1980 cohort gene expression profiles and clinical information were collected from ArrayExpress (https://www.ebi.ac.uk/biostudies/arrayexpress). The same was fetched for the RECA-EU cohort from the ICGC website (https://dcc.icgc.org/). The E-MTAB-1980 and the RECA-EU cohorts were used to validate the prognostic model’s feasibility.

GSE178481 samples were processed using the Python package “Scanpy” (Wolf et al., 2018). All samples were compartmentalized into “Tumor” and “Adjacent Tissue” groups, with filters applied to exclude cells exceeding 10% mitochondrial gene ratio, under 500 Unique Molecular Identifiers (UMIs), and with less than 250 detected gene types. The “Scrublet” Python package was used for double-cell recognition (Wolock et al., 2019). After normalization and filtering for highly variable genes, Principal Component Analysis (PCA) was undertaken using the top 50 PCs for the proceeding analysis, also addressing batch effect correction using Harmony (Korsunsky et al., 2019). The KNN neighborhood graph was constructed, followed by application of the Leiden algorithm for cluster computation, set at a resolution = 0.5. Marker genes for different clusters were identified from prior studies (Alchahin et al., 2022) with the assistance of the automatic annotation tool “MetaTiME” (Zhang et al., 2023).

### 2.2 Identification of malignant cells

We utilized large-scale chromosomal copy number variations (CNVs) to further pinpoint malignant cells. This was implemented using the R package, “inferCNV” (Puram et al., 2017), which offers a comparison between the expression spectrum across chromosomal intervals and a healthy reference. For this study, cells from normal kidney peritumoral tissues served as the normal” cell reference in deducing CNVs in suspected tumor cells. To minimize bias from specific samples, cells from the normal renal portions of multiple patients were included in the reference control group. The results from observing the CNV spectrums of tumor cells facilitated the determination of malignant cells based on their CNV scores.

### 2.3 Cell trajectory analysis

The Python package “VIA” helped us capture the evolving state and hierarchical differentiation structure of tumor cells (Stassen et al., 2021). We also explored relationships between various subclusters. This exploration was achieved by building clustering graphs, calculating likely progression paths (pseudo-time probabilities), predicting terminal states automatically, and reconstructing the lineage-based trajectory.

### 2.4 Cell communication analysis

By using the Python package “cellphoneDB” (Vento-Tormo et al., 2018), we investigated the interaction between different cell types and tumor subclusters. This process began by filtering out ligands and receptors based on their expression in each cell type, requiring that the gene be found in more than 10% of cells. We then computed the averaged expression of ligand-receptor pairs across various cell type pairs within the normalized ScRNA-seq data. The average distribution of ligand-receptor pairs was determined by randomly reshuffling cell identities in the combined data and recalculating average pair expression within 1,000 random permutations of cell identity. The significance of our observations was recorded in the numerical *p*-value, which equals the number of random pairs exceeding the observed data. We adjusted the *p*-value using the Benjamini-Hochberg method and considered a value less than 0.05 as significant. To prioritize functional ligand-receptor pairs, we assigned interaction scores. This required that both ligands and receptors show high expression in respective cell types. For comparison, we used other cell types as references.

### 2.5 Identification of DEGs

We identified differentially expressed genes (DEGs) by utilizing the R package “DESeq2”. This allowed us to discriminate between the disease-infected and normal control sets within the TCGA-KIRC cohort, as well as groups according to median risk scores. A |Log2 Fold Change (Log2FC) |> 1 and *p*-value <0.05 indicated TCGA-DEGs and RISK-DEGs. Also, using the Wilcox test, we examined variations between cancer cells and correlated peritumoral tissues from the GSE178481 database, identifying significant differences as SC-DEGs.

### 2.6 Identification of Co-expression modules

We implemented high-dimensional weighted gene co-expression network analysis (hdWGCNA) on scRNA-seq data and used SC-DEGs genes to create a hdWGNCA object (Morabito et al., 2023). This object was then transformed into a metacells object for further study. Using a soft-thresholding power of 5, we developed a co-expression network and identified crucial module eigengenes (MEs) for further analysis.

### 2.7 Protein-protein interaction analysis

The PGC1α co-expression gene module was investigated for potential protein-protein interactions using the STRING database.

### 2.8 Enrichment analysis

We utilized the R package “ClusterProfiler” for conducting Gene Ontology (GO) and Kyoto Encyclopedia of Genes and Genomes (KEGG) analyses on RISK-DEGs and MEs (Yu et al., 2012). This helped to identify the highest impact enrichment pathways and biological processes linked to the DEGs. Finally, we employed the “gsva” R package to score malignant cells concerning these identified enrichment pathways and biological processes (Hänzelmann et al., 2013).

### 2.9 The construction and validation of the prognostic model

We initially identified the overlap between module eigengenes (MEs) and TCGA-DEGs, referring to these as candidate genes. In the training set, these candidate genes underwent univariate Cox proportional hazards regression analysis. This allowed us to isolate genes demonstrating prognostically significant traits. From these, we selected variables with a *p*-value less than 0.05 for inclusion into a LASSO regression analysis, executed *via* the “glmnet” R package (Friedman et al., 2010). The purpose of the LASSO analysis was to significantly reduce the number of genes for ease of calculation. Subsequently, we designed a prognostic model with the reduced gene numbers using the formula: Risk Score = Gene one expression × coef1 + Gene two expression × coef2 + … + Gene n expression × coefn (In this formula, “expression” represents gene expression level, and “coef” denotes coefficient of the corresponding LASSO regression). This prognostic model was then evaluated for its predictive accuracy using Receiver Operating Characteristic (ROC) curves in predicting 1-year, 3-year, and 5-year overall survival rates in KIRC patients (Hebert et al., 2003). For this, we used the “survROC” package. The validity of the prognostic model was then further tested using external validation sets from the E-MTAB-1980 cohort and RECA-EU cohort.

### 2.10 Subclinical feature analysis

We categorized samples with various clinical pathologic features into subtypes, including age (either >65 years or ≤65 years), gender, pathologic M, pathologic T, pathologic stage, and OS status. Each subtype was further divided into two groups based on the median risk score: high-risk and low-risk. We assessed the variance in clinical pathologic features between these subgroups using the pairwise-Wilcoxon test or Wilcoxon rank-sum test. In order to gain a deeper understanding of the correlation between clinical pathological features and survival rate, survival analysis based on the same subclinical group was conducted for the two risk groups.

### 2.11 Independent prognosis analysis

We employed the Univariate Cox regression model to assess the accuracy of risk score evaluations and establish the influence of various clinical characteristics on patients’ prognosis. The Multivariate Cox regression model was used identifying independent prognostic factors for ccRCC patients with STED2 mutation. Utilizing these factors, we developed a nomogram model using the “cph” operation in R software. This model visually predicts the possible survival rates for patients at 1-year, 3-year, and 5-year. To confirm the model’s effectiveness, we relied on calibration curves and ROC curves to validate its precision and reliability.

## 3 Result

### 3.1 Identification of single-cell data

**FIGURE 1:**
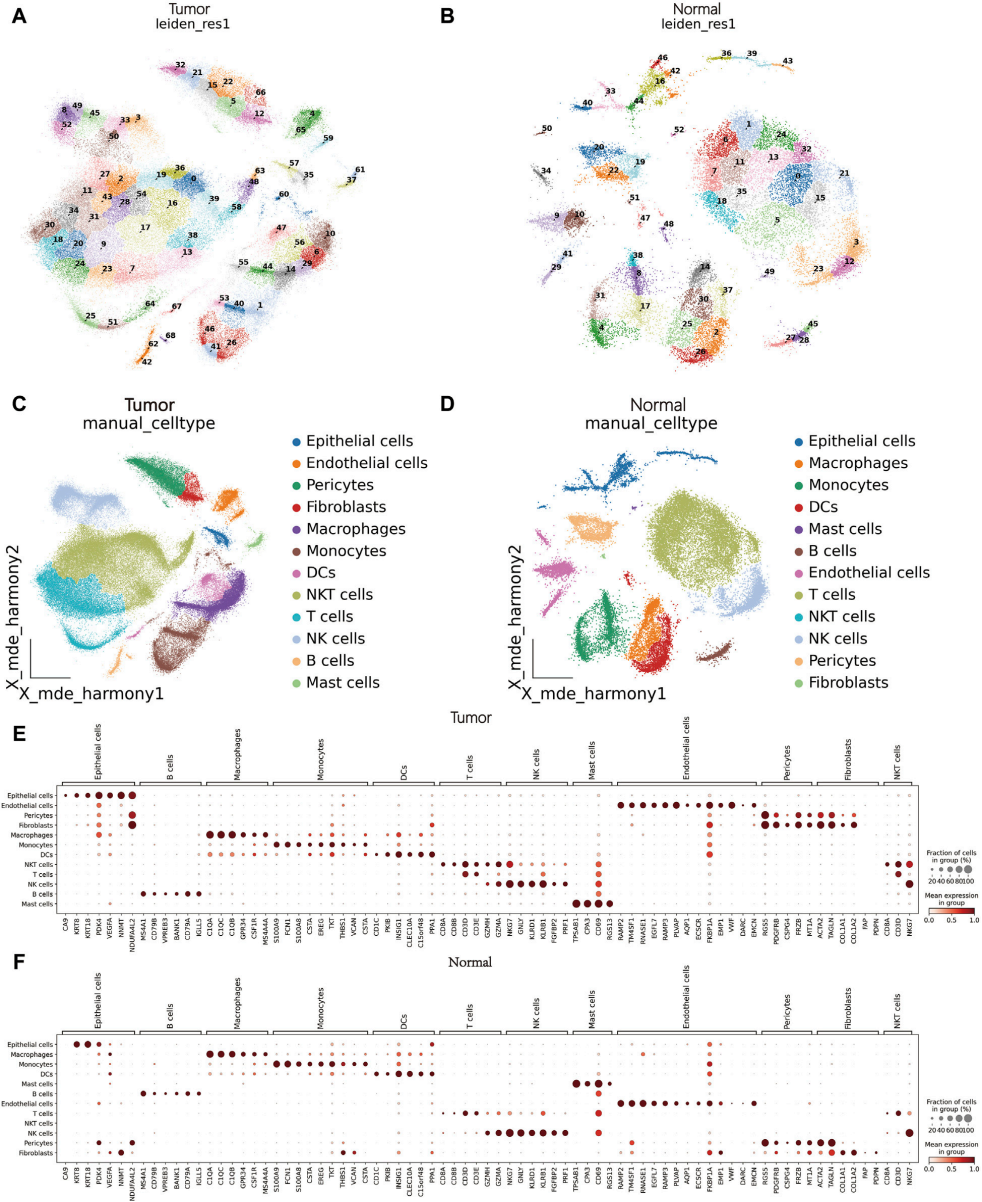
scRNA-seq data processing and annotation. **(A)** Cluster plot of tumor tissues. **(B)** Cluster plot of matched peritumoral tissues. **(C)**: Cell annotation of tumor tissues. **(D)** Cell annotation of matched peritumoral tissues. **(E, F)** Bubble chart of marker genes in tumor tissues and matched peritumoral tissues.

Initially, as detailed in the methods, we obtained clinical data of nine patients (Supplementary Table S1). A total of 114,053 and 32,016 cells were collected from tumor and peritumoral tissue groupsrespectively underwent a quality check. Logarithmic normalization was conducted, and the top 3,000 highly variable genes were used for PCA dimension reduction (Supplementary Figure S1A, B). Harmony was performed on the samples (Supplementary Figures S1C, D), and visualization was done using Uniform Manifold Approximation and Projection (UMAP) (Supplementary Figures S1E, F). Following a resolution of 0.5, they were divided into 69 and 53 cell clusters respectively (Figures 1A, B). Reference literature was used to find marker genes to manually annotate different cell clusters, with the assistance of the Python package MetaTime (Supplementary Figures S1G, H; Supplementary Table S2). Consequently, 12 cell clusters were identified including endothelial cells, mast cells, fibroblasts, pericytes, T cells, Natural Killer cells (NK), Natural Killer T cells (NKT), B cells, monocytes, Dendritic cells (DCs), macrophages, and epithelial cells (Figures 1C, D). Bubble charts were utilized to illustrate the expression of crucial marker genes across assorted cell types. (Figures 1E, F).

### 3.2 Identifying malignant cell clusters

**FIGURE 2:**
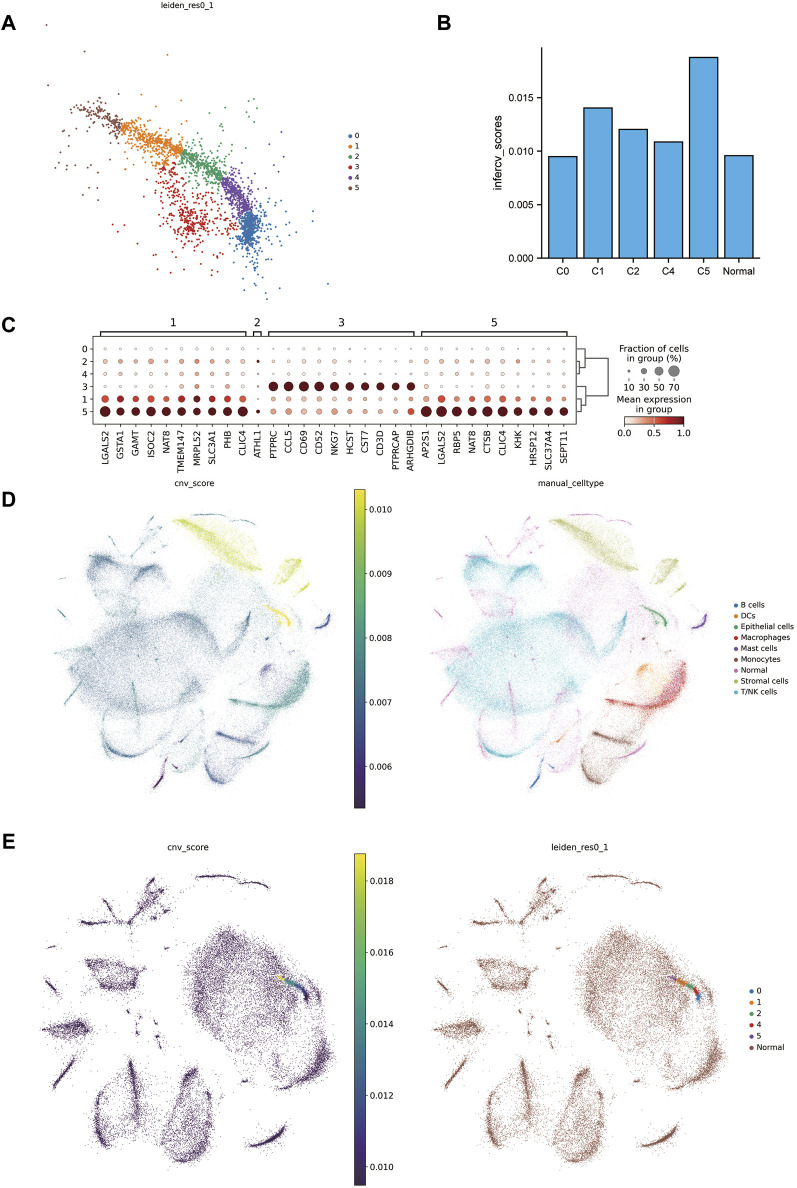
Excavation of malignant tumor cells. **(A)** Subcluster map of tumor epithelial cells. **(B)** cnv scores histogram of the subclusters of tumor epithelial cells with matched peritumoral tissues. **(C)** Marker genes of tumor epithelial cell subclusters. **(D)** On the left is the combined cnv scores distribution map of tumor tissue and matched peritumoral tissue, and on the right is the combined annotation map. **(E)** On the left is the cnv scores distribution map of tumor epithelial cell subclusters merged with matched peritumoral tissue, and on the right is the combined annotation map.

In further identify the tumor, our initial step involved sub-clustering the epithelial cell clusters of the tumor group into six distinct clusters (Figure 2A). Notably, one subcluster (C3) expressed NKT immune cell gene markers (Figure 2C) to a high degree, leading us to exclude C3 from subsequent steps. We then utilized infercnv to infer and score the chromosomal copy number variation in the tumor group cells and tumor cell subclusters, with peritumoral tissue serving as a reference (Figures 2D, F). A key finding was that mutations in ccRCC were primarily focused on the loss of chr3p and chr6, along with a gain of 5q (Supplementary Figure S2), which is in alignment with previous findings (Bi et al., 2021). We also observed the highest CNV scores in epithelial cells, further affirming the identification of the tumor tissues. Moreover, the CNV scores of the 5th subclusters (C5) were significantly increased, indicating a notable difference compared to the other subclusters and reference cells (Figure 2B).

### 3.3 Tumor subcluster development trajectory analysis and biological function differences

**FIGURE 3:**
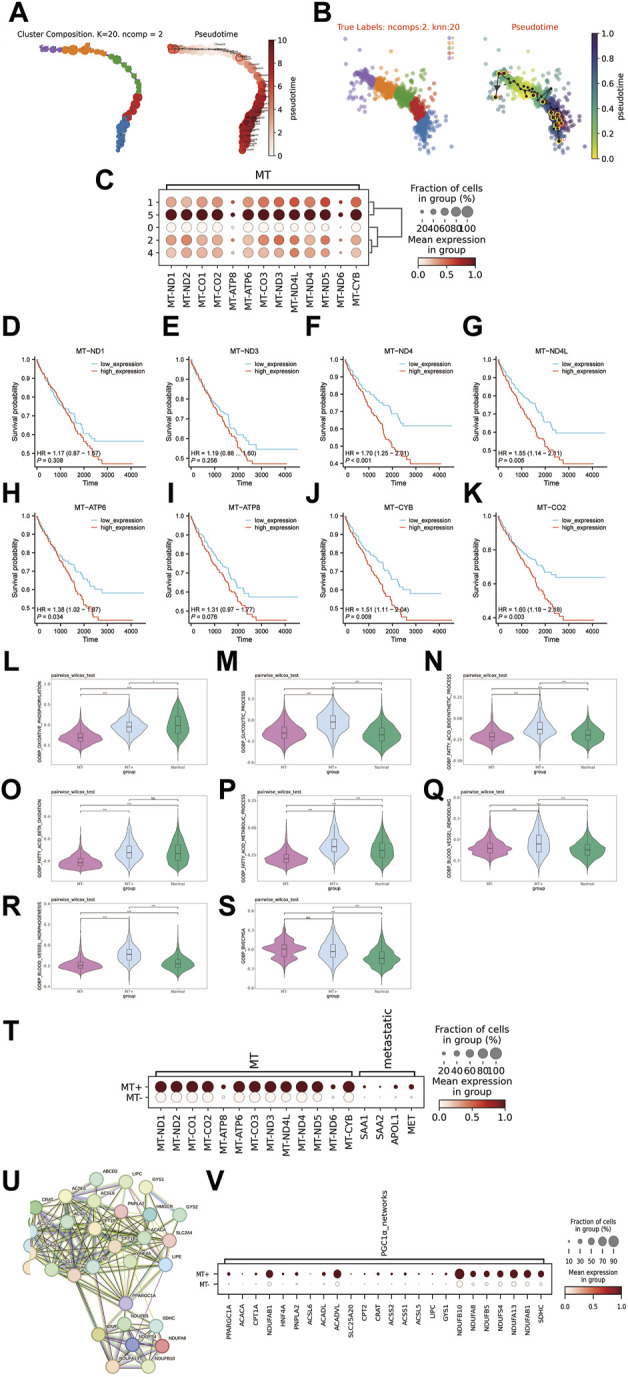
Exploring the features of the MT + group. **(A, B)** Distribution map and pseudotime trajectory of tumor cell subclusters. **(C)** Expression of mitochondrial-encoded protein genes in tumor cell subclusters **(D–K)**: Survival curves for high and low expression groups of mitochondrial-encoded genes in the TCGA-KIRC cohort (532 cases). **(L–S)**: Differences in various biological processes between MT + group, MT-group, and matched peritumoral tissue. **(L)**: Oxidative phosphorylation, **(M)**: Glycolytic process, **(N)**: Biosynthetic process of fatty acids, **(O)**: Beta oxidation of fatty acids, **(P)**: Metabolic process of fatty acids, **(Q)**: Remodeling of blood vessels, **(R)**: morphogenesis of blood vessels, **(S)**: blood vessels endothelial cell proliferation involved in sprouting angiogenesis (BVECPISA). **(T)**: Expression of mitochondrial encoded protein genes and metastasis-associated genes in MT + group and MT-group. **(U)**: PGC1α gene co-expression module, PRARGC1A is the PGC1α gene. **(V)**: Expression of PGC1α gene co-expression module in MT + group and MT-group.

To further characterize the cellular differentiation developmental trajectory withintumor cell subclusters, the cell trajectory was inferred by VIA. The results indicated two developmental directions within tumour subclusters, initiating from C1 and progressing towards C5 and C0 respectively (Figures 3A, B). Additionally, we observed that the C5 subcluster exhibited higher expression of genes encoding mitochondrial proteins compared to other subclusters (Figure 3C). Owing to the unique biological characteristics of C5, we termed it as the MT + group, while the other groups (C0, C1, C2, C4) were classified as the MT-group. Of note, higher expression of MT genes in the TCGA-KIRC cohort was significantly correlated with poor prognosis (Figures 3D–K). In terms of energy metabolism, we were astonished to discover that the MT + group exhibited efficient oxidative phosphorylation, higher when compared to the MT-group, whereas both MT+ and MT-groups had higher levels of glycolysis compared to the peritumoral tissues (Figures 3L, D–K). Furthermore, the MT + group also exhibited enhanced biological synthesis and β oxidation degradation processes of fatty acids (Figures 3N–P). The MT + group exhibited a significant improvement in angiogenesis (Figures 3Q–S). Importantly, the gene set (SAA1, SAA2, APOL1, and MET) that characterizes metastatic ccRCC from previous studies exhibited significant expression in the MT + group (Alchahin et al., 2022) (Figure 3T). Past research has illustrated that SETD2-mutated ccRCC demonstrates noticeably heightened oxidative phosphorylation and glycolysis capabilities. These characteristics align with the MT + group, and also the features found within the PGC1α gene co-expression module accord with the MT + group (Liu et al., 2019) (Figures 3U, V). Therefore, it is plausible to infer that the MT + group may have its origins in SETD2-mutated ccRCC. Collectively, we identified a subcluster of cells with high expression of mitochondrial-encoded protein genes that originate from SETD2-mutated ccRCC. The subcluster exhibits unique energy metabolism attributes, as well as high metastatic potential and poor survival prognosis.

### 3.4 Cell interaction analysis

**FIGURE 4:**
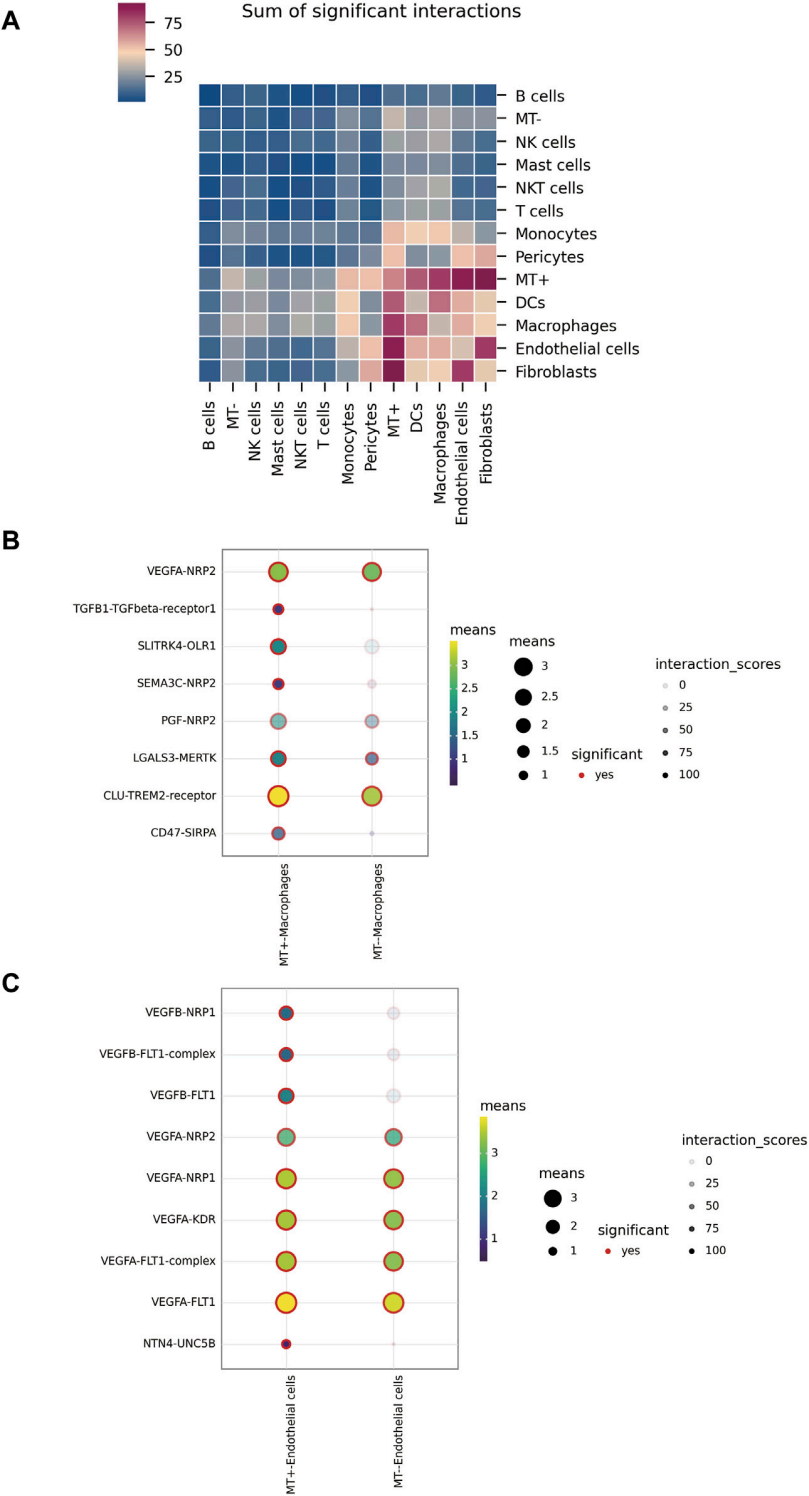
Intercellular communication. **(A)** Overall level of interaction among various types of cells in tumor tissue. **(B)** Interactions between MT + group, MT-group, and macrophages. **(C)** Interactions between MT + group, MT-group, and endothelial cells.

Our analysis with cellphoneBD revealed noteworthy interactions between the MT + group and other cells, including fibroblasts, endothelial cells, macrophages, and DCs (Figure 4A). A closer look at the interactions between the MT groups and macrophages showed distinct patterns. The MT-group displayed interactions involving crucial pairs such as CLU-TREM2, LGALS3-MERTK, PGF-NRP2, and VEGFA-NRP2. However, the MT + group demonstrated emergent interactions like TGFB1-TGFBR1, SLITRK4-OLR1, SEMA3C-NRP2, and CD47-SIRPA, going beyond those observed in the MT-group (Figure 4B). Investigating communication patterns between the MT groups and endothelial cells revealed that the MT-group primarily utilized VEGFA to interact with receptors like FLT1 (VEGFR-1), KDR (VEGFR-2), NRP1, and NRP2. However, the MT + group not only used VEGFA but also secreted VEGFB to establish contact with FLT1 and NRP1. Notably, a separate interaction involving NTN4-UNC5B was identified in the MT + group (Figure 4C). To conclude, the MT + group demonstrated a dynamic plasticity of cellular communication. They retained and expanded upon the existing modes of interaction seen in the MT-group, thereby crafting unique interaction pathways.

### 3.5 Identification of MT + related co-expression network

**FIGURE 5:**
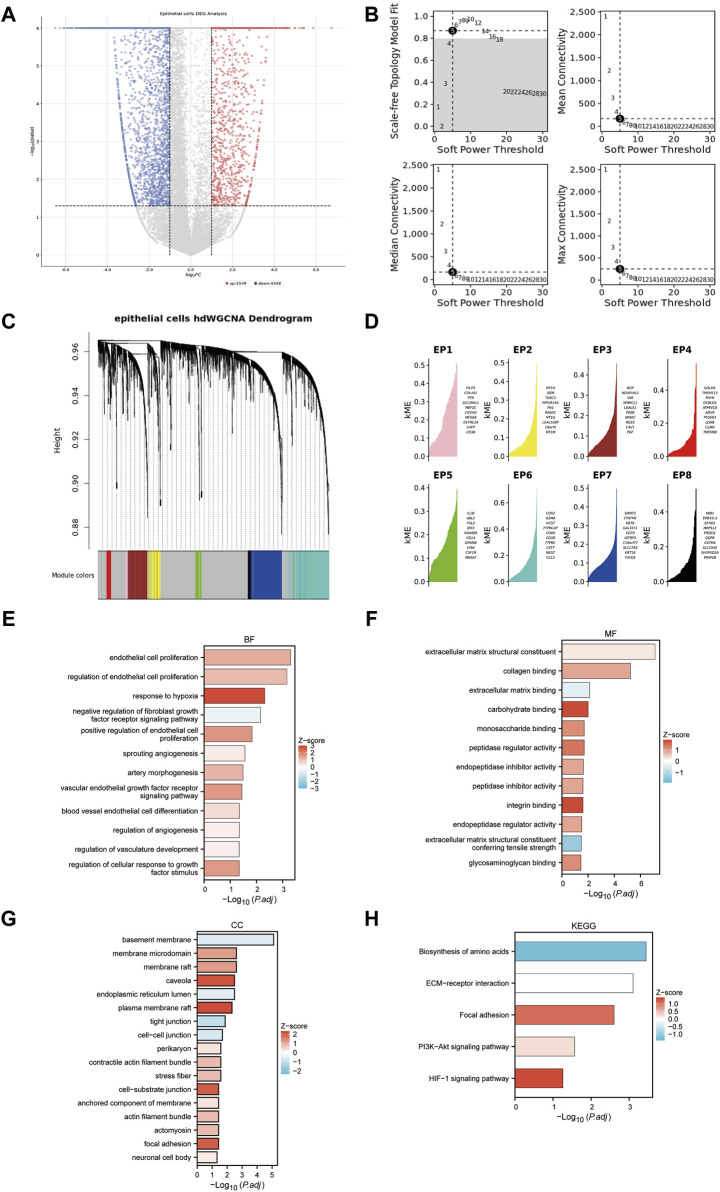
Identifying the co-expression modules. **(A)** Volcano plot of differentially expressed genes in epithelial cells of tumor samples and matched peritumoral samples. **(B)** Soft-power screening of hdWGCNA. **(C)** Hierarchical clustering tree of hdWGCNA. **(D)** eight modules from hdWGCNA clustering, each displaying the top 10 genes. **(E–H)**: GO and KEGG enrichment of the EP3 module.

Epithelial cells from tumor samples and matched peritumoral samples of GSE178481 identified 5,782 significant DEGs, referred to as SC-DEGs (Figure 5A). Using hdWGCNA method, we identified co-expression relationships among the distinct genes in SC-DEGs. We ensured the scale-free topology characteristics of this co-expression network to maintain network authenticity. Striving for maximum interconnectivity among the genes in the network, we opted for a soft power of 5. This approach established a model fitting degree for our scale-free topology model over 0.8 (Figure 5B). Our methodology led to the successful identification of eight unique gene co-expression clusters (Figure 5C). The ten genes with the highest correlation within each of these modules are listed (Figure 5D). One pivotal observation to highlight is that the third gene cluster, known as EP3, primarily displays enrichment in several biological processes. These processes encompass the regulation of the extracellular matrix and vascular formation, both intimately linked to MT + characteristics (Figures 5E–H). For this reason, we included the EP3 module in our further analysis.

### 3.6 Risk scores construction

**FIGURE 6:**
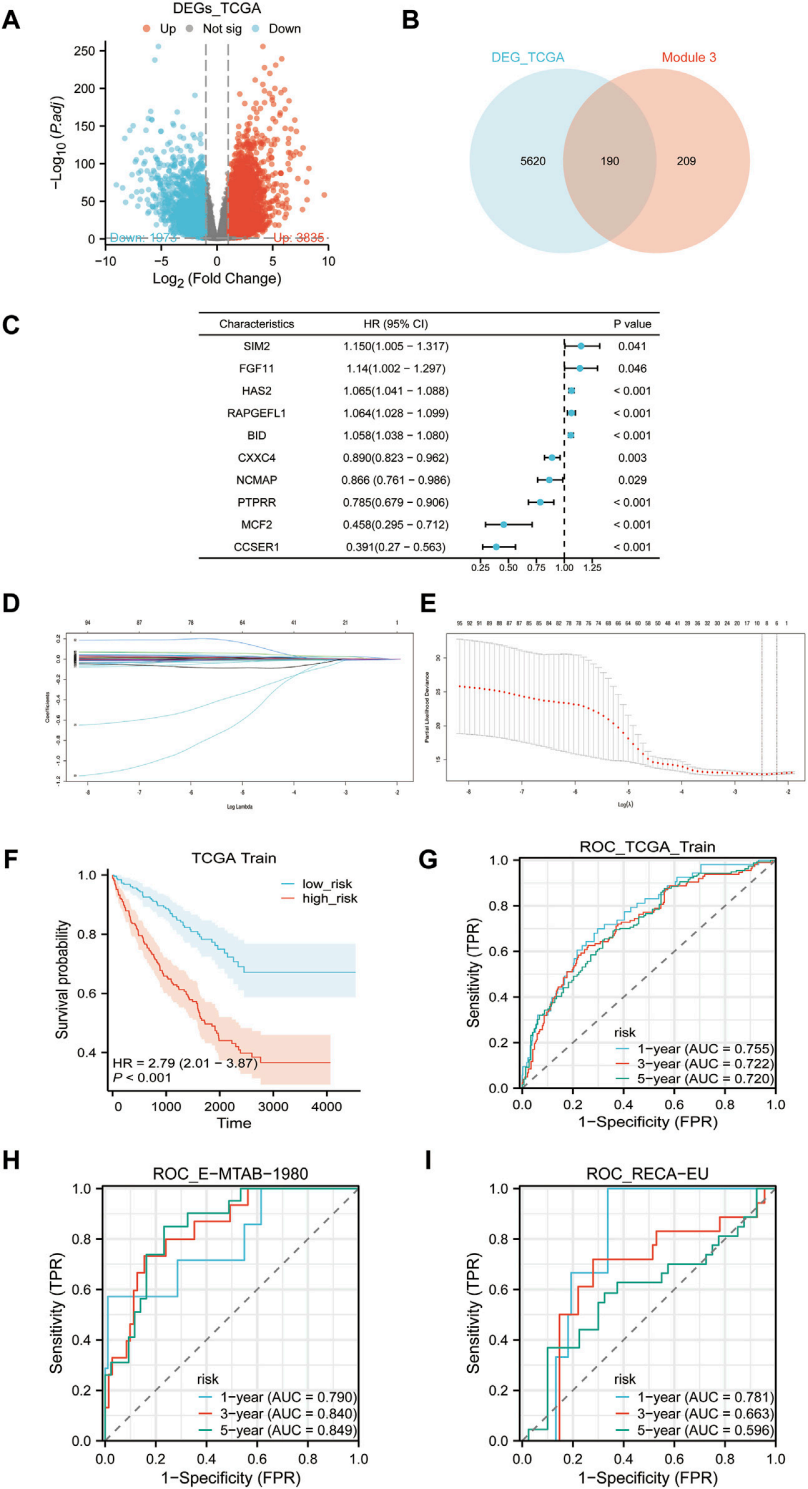
Risk scores construction. **(A)** Volcano plot of differentially expressed genes (TCGA-DEGs) between tumor samples and normal samples in TCGA-KIRC cohort. **(B)** The intersection of TCGA-DEGs and the EP3 module yields 190 candidate genes. **(C)** Univariate Cox regression analysis of the 190 candidate genes with the first five genes displayed with the biggest and smallest HR. **(D)** Lasso regression variable trajectory. **(E)** Lasso regression coefficient screening. **(F)** Survival analysis of high-risk and low-risk groups based on the median risk score. **(G–I)**: ROC curves, **(G)** TCGA-KIRC cohort, **(H)** E-MTAB-1980 cohort, **(I)** RECA-EU cohort.

Our analysis of differential gene expression used TCGA-KIRC data to identify 5,810 significantly DEGs, which we have termed TCGA-DEGs (Figure 6A). We then refined this list of TCGA-DEGs by selecting those with |logFC2|≥1, and intersecting with EP3 module genes, resulting in 190 candidate genes (Figure 6B). Utilizing the TCGA-KIRC data as a training set, we applied univariate Cox regression analysis to pinpoint 494 genes significantly correlated with OS (Figure 6C). Upon further examination in the univariate regression analysis, we selected genes with a *p*-value less than 0.05 and subjected them to Lasso regression. This resulted in six primary genes: FKBP10, LGALS1, BID, FBXO21, TEK, ACHE. Their corresponding risk scores are: 0.0001016149* FKBP10 + 0.0000405782* LGALS1+ 0.0051667850* BID -0.0012015705* FBXO21 -0.0044925698* TEK+ 0.0014603659* ACHE (Figures 6D, E). We then charted the survival curve and time-dependent ROC curve, demonstrating the appropriateness and efficiency of these risk scores with notable predictive value within the TCGA-KIRC cohort (Figures 6F, G). Additionally, our model’s time-dependent ROC curve was applied to the E-MTAB-1980 and RECA-EU cohorts, suggesting that these risk scores may be generalized to other ccRCC populations (Figures 6H, I).

### 3.7 Analysis of prognostic model and different clinical features

**FIGURE 7:**
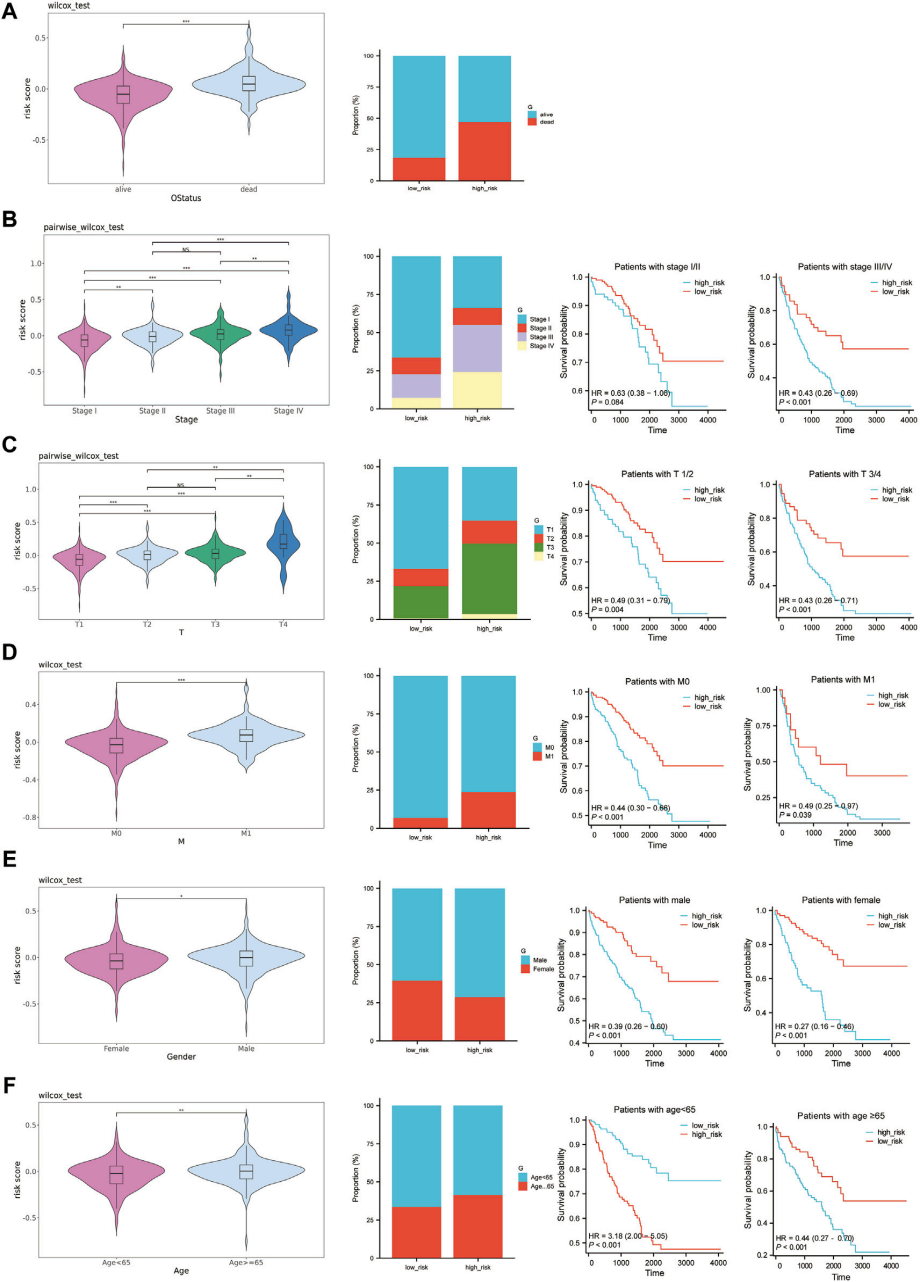
Prognostic value of risk scores in ccRCC. **(A–F)**: The left graph shows the distribution of risk scores in different clinical traits, the middle graph shows the distribution of different clinical traits in the high-risk and low-risk groups, and the right graph shows the survival curves of the high-risk and low-risk groups in the same clinical trait. **(A)** OS status. **(B)** Pathologic Stage. **(C)** Pathologic T. **(D)** Pathologic M. **(E)** Gender. **(F)** Age.

We analyzed the correlation between established risk scores and various clinical features. To do this, we compared risk score differences in patient groups differentiated by specific clinical characteristics. Our data indicated that patients falling under the M1, stages III-IV, and T3-T4 typically demonstrated higher risk scores. However, the relationship between risk score alterations and age or gender was less pronounced (Figures 7A–F). To build upon this, we conducted stratified analysis using these clinical traits. The survival analysis, conducted among patients of the same age, gender, pathologic M, pathologic stage, and pathologic T, but with different risk score levels, revealed substantial statistical significance (Figures 7A–F). Given these findings, we conclude that risk scores based on the six feature genes correlate closely with clinical features and hold applicability across various clinical conditions.

### 3.8 Construction of a nomogram

**FIGURE 8:**
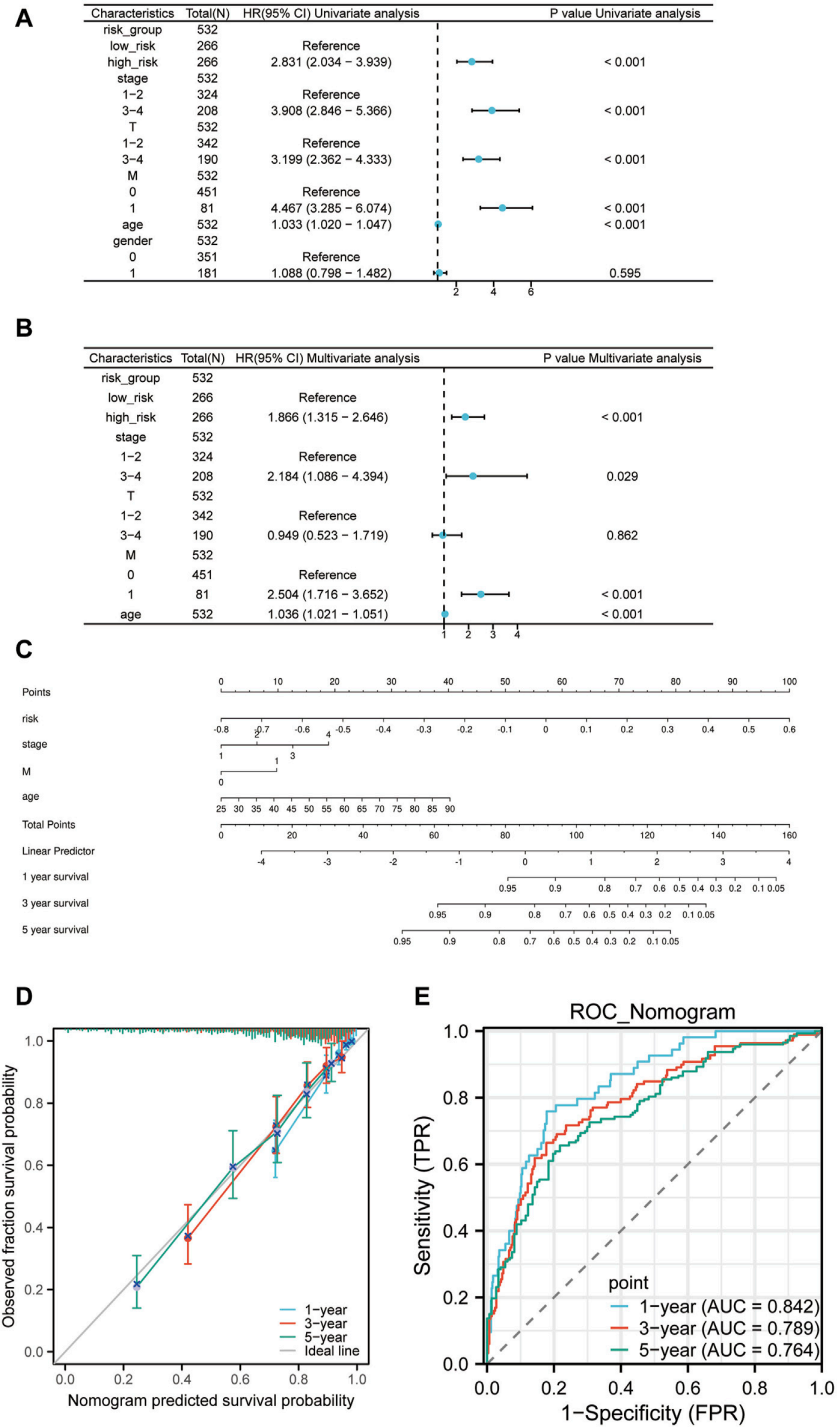
The Nomogram construction. **(A)** Univariate Cox regression of risk scores and clinical features. **(B)** Multivariate Cox regression of risk scores and clinical features. **(C)** Nomogram constructed based on the four independent prognostic factors: risk scores, pathologic Stage, pathologic M, and age. **(D)** Prognostic calibration curve of the nomogram. **(E)** Time-dependent ROC curve of the nomogram.

To sieve out independent prognostic factors, univariate and multivariate Cox analyses were conducted for clinical features and risk scores. We discovered that the risk scores, pathologic Stage, pathologic M, and age serve as independent prognostic factors for the patients (Figures 8A, B). We then constructed a nomogram model using the four independent prognostic factors (Figure 8C). Moreover, calibration curves and time-dependency ROC curves showcased that this nomogram model holds substantial predictive value (Figures 8D, E). So overall, it suggests that this specific prognostic model can be an effective tool for predicting patient outcomes based on the four independent prognostic factors.

### 3.9 Enrichment analysis between high and low-risk groups

**FIGURE 9:**
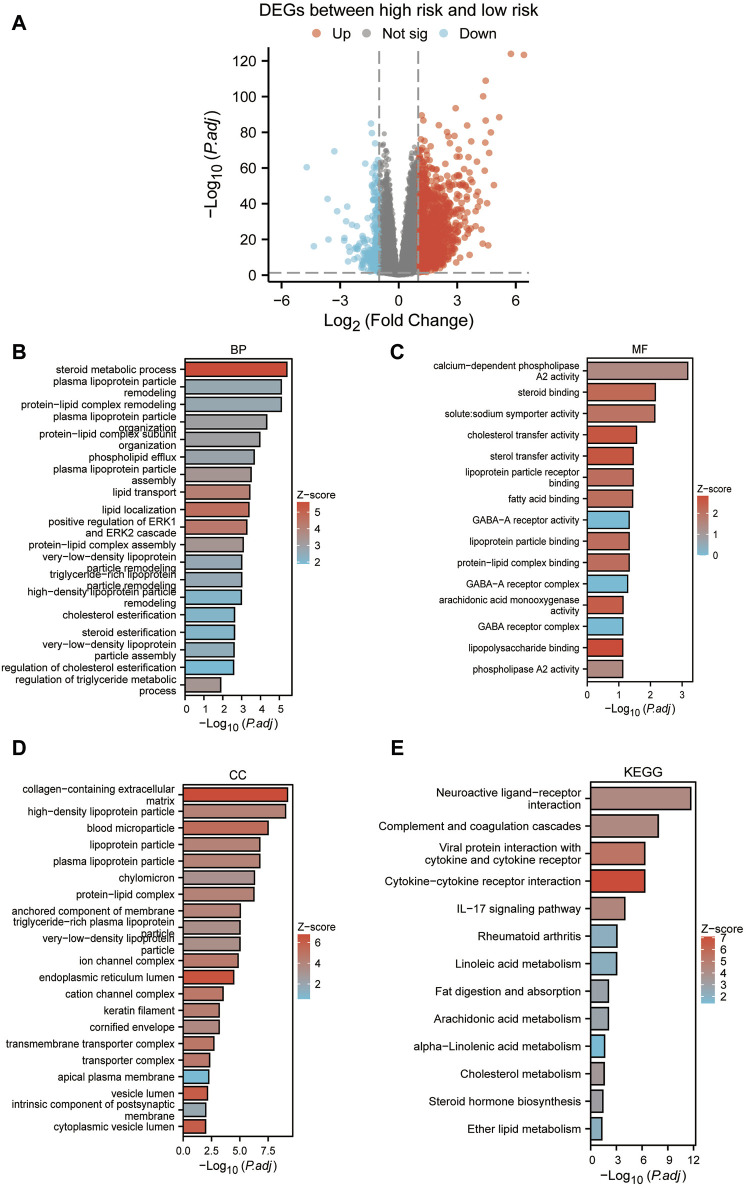
Enrichment analysis between high and low-risk groups. **(A)** Volcano plot based on the differential analysis between high-risk and low-risk groups. **(B–D)**: GO enrichment analysis. **(E)** KEGG enrichment analysis.

Based on the median risk scores, we stratified our sample into high-risk and low-risk cohorts, to elucidate the impact of varied risk levels on the progression of cancer. Consequently, RISK-DEGs were identified for further investigation (Figure 9A). An enrichment analysis was subsequently conducted to identify the most pivotal enriched pathways differentiating the two groups. The results displayed that GO was mainly enriched in lipid metabolic processes such as plasma lipoprotein, cholesterol, triglycerides, phospholipids, etc (Figures 9B–D). KEGG was mainly enriched in neuroactive ligand-receptor interaction, complement and coagulation cascades, cytokine-cytokine receptor interaction, IL-17 signaling pathway, *etc.* (Figure 9E).

## 4 Discussion

ccRCC, a widely prevalent malignant tumor, continues to show a high mortality rate despite the improved overall survival in recent decades (Siegel et al., 2020). This suggests the urgent need for in-depth analysis of ccRCC heterogeneity to better understand its pathophysiology. In earlier studies on SETD2-mutated ccRCC, the primary focus has been on epigenetic elements and cell line expriments. However, there remains a significant gap in our knowledge concerning the real-world biological manifestation of this mutation and how it interacts within the tumor microenvironment in actual patients. This study utilizes scRNA-seq to conduct an extensive investigation into ccRCC’s cellular heterogeneity, identifying twelve fundamental cell types, including B cells, endothelial cells, T cells, Natural Killer cells, Natural Killer T cells, monocytes, mast cells, DCs, fibroblasts, pericytes, macrophages, and epithelial cells. Through gene CNV and cell development trajectory analysis, ccRCC was classified into 6 subclusters. However, the subcluster C3 was excluded due to the overexpression of TNK-related marker genes. The subcluster C5, known as MT + here, exhibited unusually high expression of mitochondrial genes, separating it from the other clusters that were labeled as MT-. This high expression of mitochondrial genes in the TCGA-KIRC cohort was indicative of a shorter survival time, suggesting patients from the MT + group may face a challenging prognosis. Additionally, MT + showed distinctive energy metabolism characteristics, specifically elevated levels of oxidative phosphorylation and glycolysis─ a sharp contrast to the well-known Warburg effect (Zhu et al., 2023). Intriguingly, previous studies have found similar metabolic patterns, of increased oxidative phosphorylation and glycolysis, in SETD2-mutated ccRCC─ an unusual feature for ccRCC (Liu et al., 2019; Xie et al., 2022). This finding is substantiated by the presence of the PGC1α gene module in the MT + group, an occurrence also observed in SETD2-mutated ccRCC (Liu et al., 2019; Xie et al., 2022). This correlation suggests that the MT + group may originate from SETD2-mutated ccRCC. In addition, our findings show that MT + substantially increased both the biosynthesis and β-oxidation in lipid metabolism, which resulted in an overall upsurge in lipid metabolism. This observation departs from the prevailing understanding of ccRCC, which proposes that lipid synthesis and storage are increased, while lipid usage and oxidation are decreased (Shi et al., 2023; Zhu et al., 2023). This imbalance is believed to result in a build-up of cholesterol, fatty acids, and triglycerides. This discrepancy could be explained by the overactive expression of β-oxidation-related genes triggered by SETD2 mutation. The MT + group furthermore portrayed the expression of metastasis-related gene clusters (SAA1, SAA2, APOL1, and MET) (Alchahin et al., 2022). The expression of these metastasis-related gene clusters within the MT + group might be associated with its unique energy metabolism. Higher levels of oxidative phosphorylation and glycolysis could permit its survival and proliferation within the malignant tumor environment. This significant difference in energy metabolism could potentially enhance the invasiveness and metastatic capacity of the tumor. Aside from exhibiting enhanced survivability, the MT + group evidenced a significant capacity to induce angiogenesis, which is strongly linked to the group’s interactions with endothelial cells. This particular aspect will be discussed in further detail in the subsequent section.

In the tumor microenvironment, we observed significant interaction between MT + group and other cell types, most notably with Fibroblasts, Endothelial cells, Macrophages, and DCs. Compared to the MT-group, the MT + group possessed a stronger, wider range of cellular interactions, with many unique interaction patterns emerging. For instance, in the interaction between MT+ and Macrophages, the crosstalk between CLU-TREM2 and LGALS3-MERTK significantly increased. Prior studies indicated that TREM2 could regulate the polarization of macrophages from M1 to M2 through the NF-κB/CXCL3 axis (Fang et al., 2024; Shang et al., 2024), and MerTK mediated phagocytosis to enhance M2 polarization of macrophages (Lin et al., 2022). In addition, interactions like SLITRK4-OLR1 and TGFB1-TGFBR1 only existed in the MT + group. Previous research suggested a significant correlation between OLR1 expression and M2 cell infiltration (Sun et al., 2021), and TGFB1 could promote M2 polarization of macrophages *via* the MAPK signaling pathway (Liu et al., 2022; Maldonado et al., 2022). Moreover, our research found that activation of NRP2 could stimulate phagocytosis in macrophages and orchestrate immunosuppression to facilitate tumor growth (Roy et al., 2018; Yang et al., 2022). We also discovered that the CD47-SIRPA mediated tumor cell evasion from macrophage phagocytosis (Zhang and Fu, 2018; Li et al., 2022). When interacting with Endothelial cells, MT-secretion of VEGFA could bind to FLT1(VEGFR-1) and KDR (VEGFR-2), inducing angiogenesis, as well as vascular remodeling and enlargement (Pérez-Gutiérrez and Ferrara, 2023; Shaw et al., 2024). However, MT + not only secreted VEGFA but also VEGFB. In cancers, VEGFB is reported to be capable of promoting cancer metastasis *via* non-VEGF-A-dependent mechanisms (Yang et al., 2015; Shaw et al., 2024). Additionally, NRP1 and NRP2 functioned as auxiliary receptors of VEGF to amplify signal transduction (Pérez-Gutiérrez and Ferrara, 2023; Shaw et al., 2024). Particularly, the interaction between NTN4-UNC5B in the MT + group could attract endothelial progenitor cells (EPC) to migrate to the site of vascular injury to facilitate angiogenesis and tissue repair (Lee et al., 2020). These findings suggested that MT + possesses a stronger angiogenesis-inducing ability and metastatic potential. Collectively, MT + possesses stronger and more versatile cellular interactions, which is especially pronounced in its crosstalk with Macrophages and Endothelial Cells. MT + presents the capability of inducing M2 cells, inhibiting macrophage immunity, promoting tumor angiogenesis, and increasing its metastatic potential.

In renal cancer, there are two other common subtypes, characterized by PBRM1 and BAP1 mutations. Previous studies report a sequential relationship between PBRM1 and SETD2 mutations and reveals an exclusionary relationship with BAP1 mutations (Peña-Llopis et al., 2013). The PBRM1 mutation largely promotes the development and progression of ccRCC by down-regulating HIF-1α signal transduction (de Cubas and Kimryn Rathmell, 2018). On the other hand, the BAP1 mutation leads to an increase in glycolysis and a reduction in mitochondrial respiration (Dai et al., 2017). In contrast, this study reveals that MT + cells with SETD2 mutations demonstrate high levels of mitochondrial respiration. This finding is consistent with the mutually exclusive relationship between SETD2 and BAP1 mutations.

To characterize the impact of MT + on patient prognosis, we used SC-DEGs to identify an MT + gene co-expression module EP3 related to angiogenesis in hdWGCNA. We then overlapped these results with TCGA-DEGs to shortlist 190 potential genes. Univariate Cox regression analysis and LASSO algorithm were used to develop a risk factor scoring model based on six prognostic genes (FKBP10, LGALS1, BID, FBXO21, TEK, ACHE). This model showed good performance in predicting the OS of KIRC patients and exhibited high predictive power in the TCGA cohort according to the ROC. In addition, we verified the predictive power of this model using two cohorts (E-MTAB-1980 and RECA-EU) with zero overlap with the TCGA cohort. The relationship between the model prediction and clinical manifestations was also investigated. Due to incomplete N staging information, it was not included as a clinical feature. Patients at stage M1, stage II to IV, and stage T3 to T4 demonstrated higher risk scores. However, the risk score had a poor correlation with patients’ age and gender. Besides, survival analysis showed significant statistical differences in patients with the same clinical features grouped by risk score. The finding indicates that the predictive value of the model not only extends to OS, but also possesses powerful prediction for clinical features. It also carries high predictability for patients with different clinical features. To enhance the predictive power of the model, we considered the impact of clinical features on the model. We carried out univariate and multivariate regression cox analysis with the risk scores and clinical features. The results suggested that risk scores, pathologic Stage, pathologic M, and age are independent prognostic factors in KIRC patients. Further, we developed a nomogram model and verified its robust prediction ability by using calibration curves and ROC curves.

Lastly, risk stratification using the model’s group median score distinguished all samples into a low-risk group and a high-risk group. We observed a high-risk group is mainly enriched in metabolic processes related to plasma lipoproteins, cholesterol, triglycerides, phospholipids, *etc.* KEGG was mainly enriched in neuroactive ligand-receptor interactions, the complement and coagulation cascades, cytokine-cytokine receptor interaction, and the IL-17 signaling pathway. Our study emphasized the close association between blood vessel formation and lipid metabolism in ccRCC.

Despite these discoveries have shed some new light on the key features and biological behaviors of SETD2-mutated ccRCC, these features and interactions require further experimental validation. Furthermore, establishing whether this prognostic model can be generalized to other types of RCC requires further investigation. Future work will involve studying key cell-cell interaction functionalities through coculture and improving and promoting the use of this prognostic model.

## 5 Conclusion

Our study uncovered crucial transcriptomic characteristics of SETD2-mutated ccRCC within an actual tumor environment. These include heightened mitochondrial gene expression and increased oxidative phosphorylation, glycolysis, lipid metabolism, and angiogenesis abilities. Comparison of gene expression and biological behavior between the MT+ and MT-groups exposed a potential for M2 cell formation in the MT + group. This is mediated by a combination of four receptors: TREM2, MERTK, OLR1, and TGFBR1. Additionally, our prognostic model can efficiently foresee the survival timeline of ccRCC patients. These discoveries reinforce and broaden prior research, which could potentially enrich our comprehension of ccRCC, foster the development of new treatment methods, and enhance prognosis assessment of ccRCC patients.

## Data Availability

The original contributions presented in the study are included in the article/Supplementary Material, further inquiries can be directed to the corresponding author.

## Abbreviations

scRNA-seq, single-cell RNA sequencing; RCC, Renal cell carcinoma; ccRCC, clear cell renal cell carcinoma; PCA, Principal Component Analysis; DEGs, differentially expressed genes; hdWGCNA, High-dimensional weighted gene correlation network analysis; OS, overall survival; VHL, von Hippel-Lindau; HIF-1, hypoxia-inducible factor-1; VEGF, factors like vascular endothelial growth factor; H3K36me3, H3K36 trimethylation; GEO, Gene Expression Omnibus; TCGA, The Cancer Genome Atlas; UMIs, Unique Molecular Identifiers; Log2FC, Log2 Fold Change; MEs, module eigengenes; NK, Natural Killer cells; NKT, Natural Killer T cells; DCs, Dendritic cells; GO, Gene Ontology; KEGG, Kyoto Encyclopedia of Genes and Genomes; ROC, Receiver Operating Characteristic.
