# Supplementary material for: Revealing the characteristics of SETD2-mutated clear cell renal cell carcinoma through tumor heterogeneity analysis
Source: Front Genet. 2024 Jul 25;15:1447139. doi: 10.3389/fgene.2024.1447139 (PMC11306021; doi:10.3389/fgene.2024.1447139)
Supplement: Supplementary file 1 [file Table3.DOCX]

**Supplementary Figure 1**

**
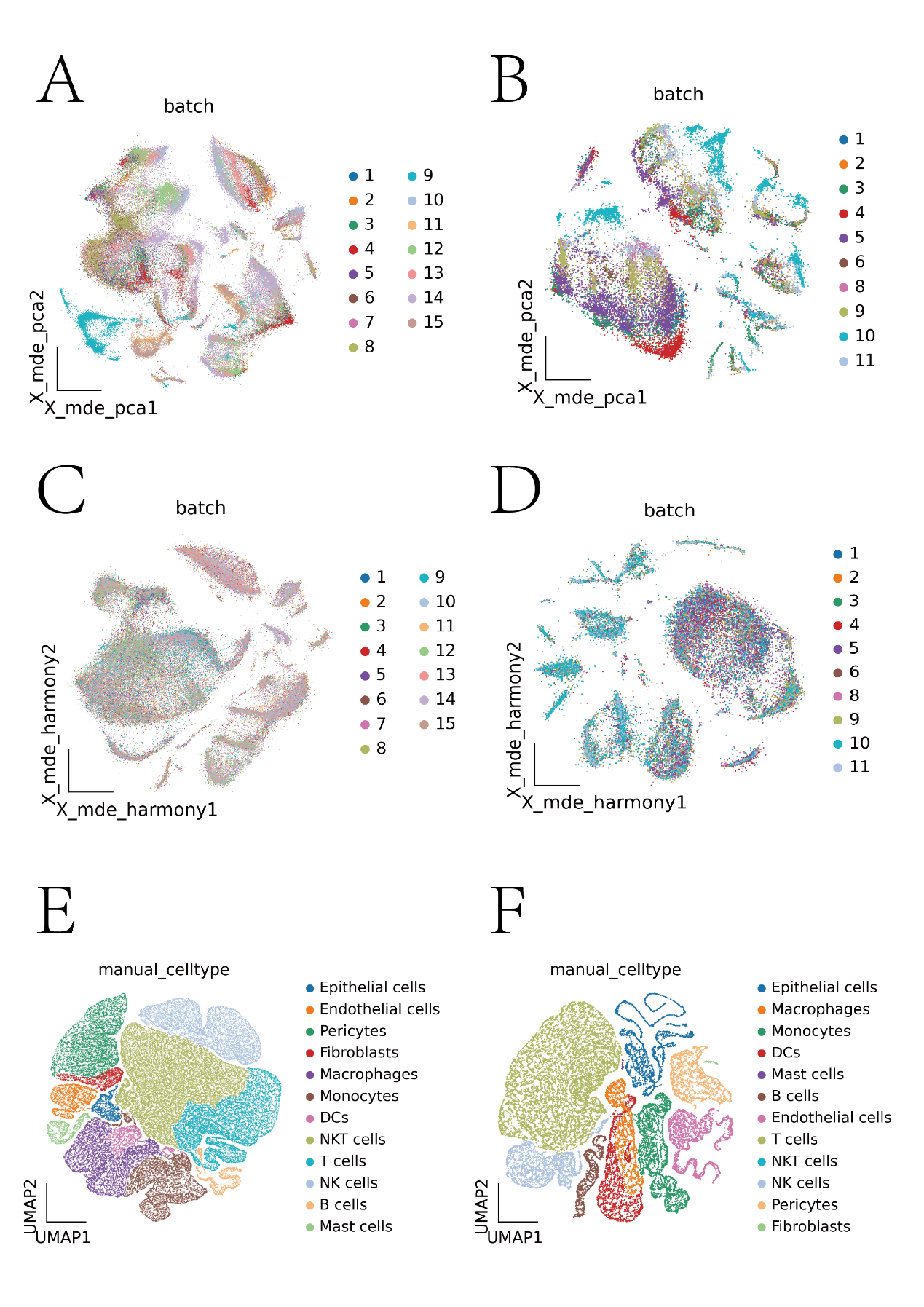
**

Supplementary Figure 1: A: PCA-dimensionality reduced distribution map of tumor tissues. B: PCA-dimensionality reduced distribution map of matched peritumoral tissues. C: Harmony-adjusted distribution map for tumor tissues. D: Harmony-adjusted distribution map for matched peritumoral tissues. E: UMAP distribution diagram of tumor tissues. F: UMAP distribution diagram for matched peritumoral tissues.

**Supplementary Figure 2**


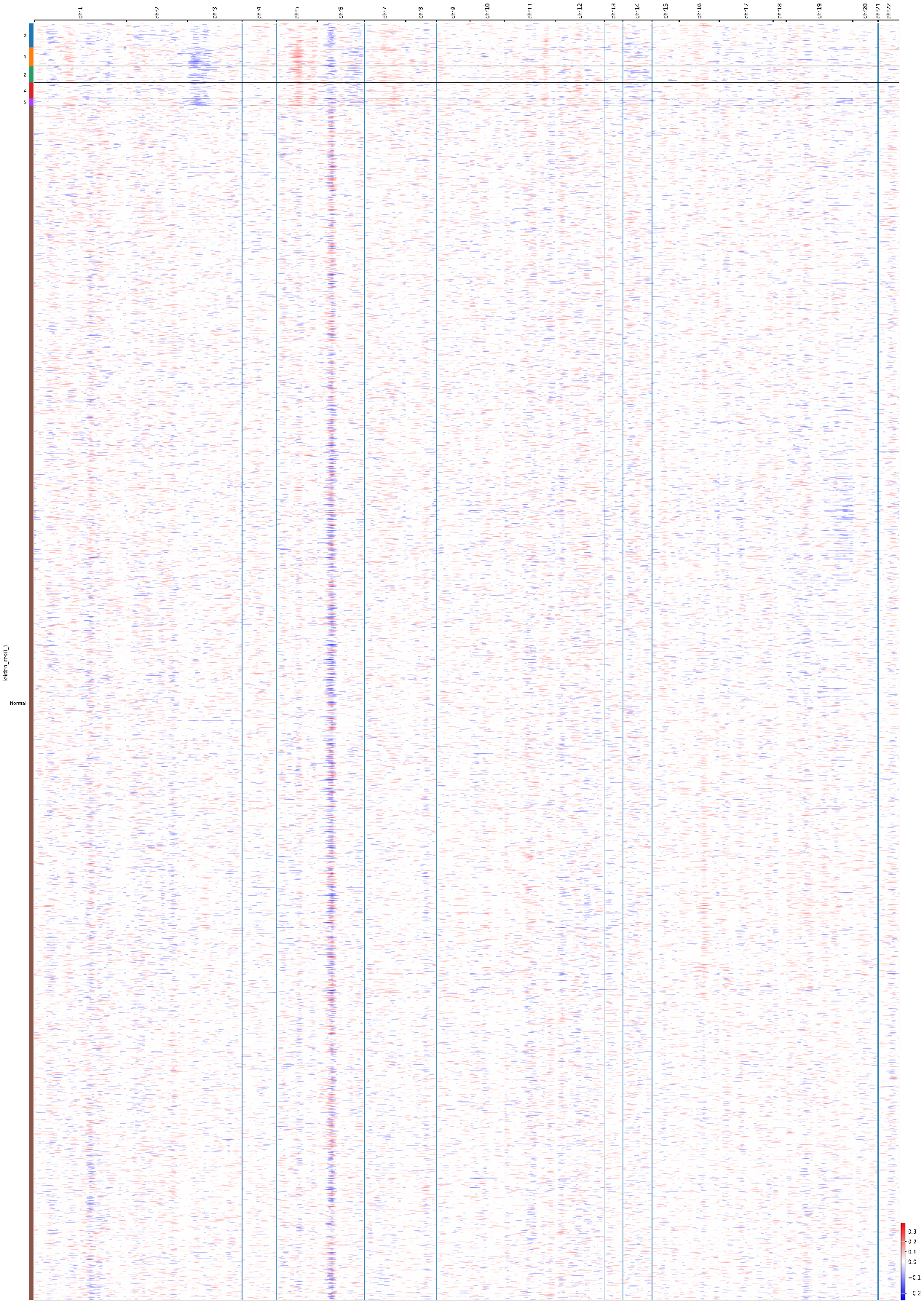


Supplementary Figure 2: In the CNV heatmap, it is observable that the loss of chr3p, chr6 and the gain of 5q are present in tumor clusters C0-C5.
